# Regional lung function in congenital diaphragmatic hernia assessed using electrical impedance tomography

**DOI:** 10.1038/s41390-025-04185-9

**Published:** 2025-06-18

**Authors:** Ellen Douglas, Kristin N. Ferguson, David G. Tingay

**Affiliations:** 1 Neonatal Research, Murdoch Children’s Research Institute, Parkville, VIC Australia; 2 Neonatology, The Royal Children’s Hospital, Parkville, VIC Australia; 3 Department of Paediatrics, University of Melbourne, Melbourne, VIC Australia; 4 Department of Critical Care, University of Melbourne, Melbourne, VIC Australia

## Abstract

**Background:**

Congenital diaphragmatic hernia (CDH) is characterised by ventilation disunity due to unilateral pulmonary hypoplasia. We aimed to describe lung function in each of the ipsilateral (hypoplastic) and contralateral CDH lungs during neonatal intensive care unit (NICU) admission.

**Methods:**

Prospective, observational study including infants with left-sided CDH. Electrical impedance tomography (EIT) images of both lungs were obtained over 20 min at three clinically distinct timepoints: pre-repair, post-repair and prior to NICU discharge. Centre of Ventilation (CoV, measure of regional distribution of tidal ventilation; <50% favours contralateral lung), aeration and time constants were calculated between the ipsilateral and contralateral lung.

**Results:**

Fifteen infants were included with median (range) gestational age 38 (34, 41) completed weeks, observed-to-expected lung-to-head ratio 51 (36, 67)% and 87% Type B or C lesion. Eight infants received high-frequency ventilation during admission. Ventilation was predominantly contralateral, with the ipsilateral lung ventilation increasing from pre-repair CoV 35 (31, 50)% to 42 (36, 49)% pre-discharge. Ipsilateral aeration was unchanged; 45 (38, 49)% of overall aeration pre-repair to 45 (33, 50)% pre-discharge. Time constants were 198 (143, 290) and 203 (141, 292) ms in the contra- and ipsilateral lung at discharge.

**Conclusions:**

Lung function changes with time in CDH, and monitoring of each lung is possible using EIT.

**Impact:**

- Respiratory support of infants born with congenital diaphragmatic hernia (CDH) is challenging due to an inability to determine the magnitude of ventilation disunity resulting from unilateral pulmonary hypoplasia.
- Electrical impedance tomography (EIT) could describe the aeration and ventilation characteristics of the ipsi- and contra-lateral lungs throughout the NICU course in 15 infants with left-sided CDH.
- Ventilation, but not aeration, in the ipsilateral lung increased from the pre-repair state by discharge.
- This suggests EIT could be used to define lung function changes with time in CDH, providing a detailed understanding of each lung, which that may assist in refining care.

## Introduction

Congenital diaphragmatic hernia (CDH) is characterised by pulmonary hypoplasia.^1–4^ The degree of pulmonary hypoplasia depends on the timing and magnitude of herniation of abdominal contents into the foetal thorax, as well as genetic and other antenatal factors.^5^ These factors exert a direct compressive effect on the lungs and also impair the coordination of lung development and growth.^6^ This results in a variable presentation of right and left lung size and function, and thus clinical respiratory failure.^5–7^ Consequently, mechanical ventilation of the CDH lung is difficult, and the risk of injury is high.^8^ Current clinical guidelines focus on minimising volume and pressure exposure, with a lower threshold for high-frequency ventilation (HFV).^3,9,10^ In part, this is because clinicians lack the sophisticated, bedside tools needed to optimise respiratory care for infants with CDH.

An often-overlooked aspect of CDH is the heterogeneity of lung growth and the potential for lung disunity as a driver for lung injury. Theoretically, each lung in CDH should have different lung mechanics, with different safe tidal volumes, opening pressures and time constants. Targeting ventilator settings that are effective and protective for one lung may be causing volutrauma or atelectasis in the other. At present, clinicians have no guide to the functional degree of lung disunity. Lung size on chest radiograph is not a reliable measure of lung volume or gas exchange.^11^ Bedside measures of respiratory function either consider lung function as a whole (such as tidal volume level and carbon dioxide clearance) or can be impacted by right and/or left ventricular dysfunction (such as oxygenation).

Electrical impedance tomography (EIT) is a non-invasive, radiation-free method of imaging dynamic volume change within the lung on a breath-by-breath basis.^12^ Unlike lung ultrasound, EIT can determine relative tidal volume, end-expiratory volume, gas flow patterns, dynamic compliance and inspiratory and expiratory time constants in different parts of the lung at the same time.^12–14^ As EIT also generates a map of gas distribution, it may also provide an image of relative lung size, allowing comparison of lung growth as well as function over time. Despite the apparent benefits of EIT in the CDH population, only two reports exists.^15,16^ The first was a case report demonstrating that EIT could detect differences in right and left lung ventilation in an infant with an unrepaired left-sided CDH. The second study used EIT to describe the response of the CDH lung to different positive end-expiratory pressure settings pre- and post-repair.^16^

The aims of our observational study were to describe the lung function, using EIT, in the ipsi- and contra-lateral lungs in left-sided CDH, and how this changed with time during the neonatal intensive care unit (NICU) admission. We hypothesised that EIT would be able to identify regional differences in aeration, tidal ventilation and time constants consistent with the expected differences in lung hypoplasia and identify changes in lung function within each lung with time and improvement in respiratory status.

## Methods

This prospective, observational study was undertaken at the Royal Children’s Hospital (RCH) NICU, Melbourne, Australia. The RCH is a regional referral NICU that manages the majority of CDH cases born in Victoria. Due to COVID-19 and staffing delays, the study was run over two periods, from August 2016 to June 2018 and from April 2021 to June 2023. The study was approved by the RCH Human Research Ethics Committee (HREC 36159A). The STROBE statement is available in the Supplementary Material.

Infants were eligible for inclusion if they had a diagnosis of left-sided CDH and were clinically stable enough to allow application of the measurement equipment. Infants were excluded if they had a diagnosis of right-sided CDH or were on a pathway to extracorporeal membrane oxygenation or palliation.

**Fig. 1:**
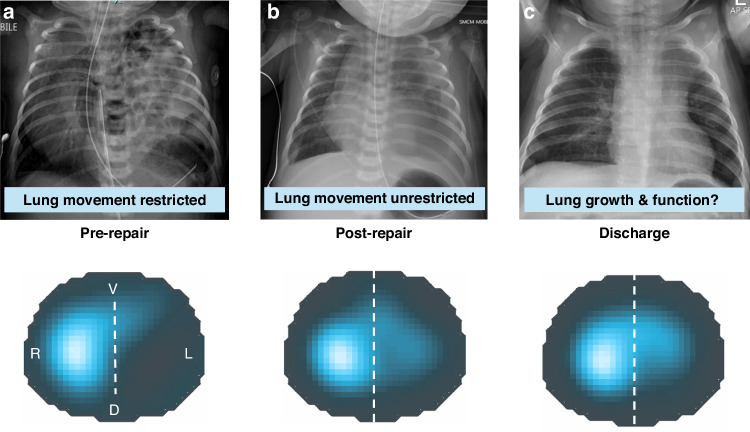
Summary of study timeline. **a** First lung imaging performed during representative respiratory support before surgery (pre-repair) to close the congenital diaphragmatic hernia (CDH). At this timepoint the left (ipsilateral) lung is hypoplastic and lung movement in both lungs (but especially the left) is restricted by hernia contents in thorax. **b** Second lung imaging performed as soon as possible (defined by clinical stability) after CDH repair (post-repair). At which point the left lung is likely to be as equally hypoplastic as in pre-repair state, but movement is not restricted by herniated contents in thorax. **c** Pre-discharge imaging undertaken at time clinical team commenced discharge planning. This timepoint was variable and defined not on absolute time from surgery but rather functional state. Lower panels demonstrate representative functional electrical impedance tomography (EIT) images (no lung region filter) during a single respiratory cycle at each timepoint.^12^ Magnitude of tidal ventilation in each lung region is expressed using a colour scale from dark blue (minimum) to light blue/white (maximum). Abbreviations: *R* right, *L* left, *V* ventral, *D* dorsal (dotted line demarcates ipsilateral [left] and contralateral [right] hemithorax).^12^

For this study, infants were studied at three clinically distinct timepoints during periods of stability for measurements as detailed in Fig. 1 and Supplementary Fig. S1: before surgical repair of the CDH (pre-repair), as soon as possible following surgical repair of the CDH (post-repair) and when the infant was not intubated, and discharge planning had commenced (pre-discharge). Infants were excluded from a timepoint if they were considered too clinically unstable to tolerate handling for placement of measurement equipment.

At each timepoint, four by 5 min continuous lung imaging and physiological measurements were made over approximately a 45 min period of stable clinical care whilst supine and on the respiratory support set by the clinical team.^17–19^ Electrical impedance was measured using LuMon EIT System (Sentec AG, Landquart, Switzerland) and the manufacturer’s non-adhesive 32-electrode neonatal EIT belt (sized to chest circumference).^20,21^ The belt was placed as high on the chest as possible, ideally directly under the armpits, to capture the hypoplastic ipsilateral (left) lung.^22^ EIT images were measured at 48 frames per second using the manufacturer’s image-acquisition software. Where the infant was ventilated, flow, pressure and tidal volume (V_T_) were measured using the Florian Respiratory Function Monitor (Acutronic Medical Systems, Hirzel, Switzerland) and simultaneously recorded using LabChart™ (AD Instruments, Sydney, Australia). Clinical staff were informed if any potential pathological event (such as air leak) was identified during the measurements. Infant demographics (gestational age, chronological age, weight and sex) and relevant clinical data (such as antenatal observed to expected lung-to-head ratio [O/E LHR], liver position, surgical intervention, sedation, analgesia, muscle relaxant, inotrope, prostaglandin and pulmonary vasodilator use) were collected.

EIT data was reconstructed using a simple chest model that defined the left and right hemithorax as equal, without pre-defined lung contouring, using the manufacturer’s software package (ibeX; Swisstom AG, Switzerland) as per standardised guidelines.^12^ Rather than applying an infant-specific chest model, this model was chosen as it makes no assumptions as to the location of the lung and heart contents within the chest. Pilot analysis suggested that the manufacturer’s infant-specific chest model may have excluded distorted lung tissue, especially in the ipsilateral lung. Recordings were manually checked for quality and presence of artefact and a band-pass filter applied to exclude the cardiac signal.^18^ Where the infant was conventionally ventilated, receiving non-invasive respiratory support or not on respiratory support, the first two minutes of stable artefact-free imaging that met imaging quality were analysed.^23^ Suitable quality data was defined as 1) no infant movement (defined by researcher at the time of measurement), 2) good belt electrode-skin contact and 3) data that contained no clear periods without an EIT tidal signal. A maximum of one minute was selected from any one of the four recordings, to ensure at least 40–60 individual consecutive lung inflations were included for analysis. Any recordings where most of the data was deemed low-quality or significantly affected by artefact were excluded. Where the infant was receiving high-frequency oscillatory ventilation (HFOV) or high-frequency jet ventilation (HFJV), ~20–30 s of data were analysed.^24^ If the infant was clinically stable, the HFJV pulse was paused for 30–60 s and conventional mechanical ventilation (CMV) at a rate of 30–40 breaths per minute was applied to generate data of conventional tidal volume changes for analysis. A clinician experienced in HFJV was present during these measurements.

Cleaned, filtered and selected data were extracted for the centre of ventilation (CoV); geometric mean of tidal volume) and time-course impedance change for the right and left lung.^12^ CoV was reported for the right (contralateral) to left (ipsilateral; CoV_RL_) and gravity-dependent (ventral to dorsal; CoV_VD_) planes. The end-expiratory lung volume (EELV) was calculated from the time-course impedance change in aeration for each region of interest.^12^ Relative lung aeration in the ipsilateral lung (left) was defined as the percentage of left chest EELV relative to the whole chest EELV.^12,25,26^ Although the right lung is known to be anatomically larger than the left, no reference for an ideal CoV along the right-left and gravity-dependent planes was applied. This is because the true anatomical lung volumes of each lung in CDH are unknown and are likely to vary between infants. Additionally, inspiratory (T_i_) and expiratory time (T_e_) and inflation time constant (τ) were calculated from 15 representative continuous breaths during the pre-discharge measurement (using LabChart peak analysis software) to determine the mechanical characteristics of the contralateral and ipsilateral lung during spontaneous breathing. All measures, except for time constants, were compared to the pre-repair lung state. Infants in whom pre-repair data were not available were excluded.

### Statistical analysis

A convenience sample of 15 infants was chosen to reflect admission rates in our unit for CDH, feasibility and previous physiological observational studies in this population.^27^ Sample sizes of 15–20 infants with normally developed lungs have been shown to demonstrate meaningful differences in CoV and aeration during different modes of support and after interventions.^18,24,28^ Descriptive statistics were used to define physiological measures at each timepoint and comparisons were made using non-parametric tests as appropriate. Data were analysed in GraphPad Prism (v9.1.2).

## Results

**Fig. 2:**
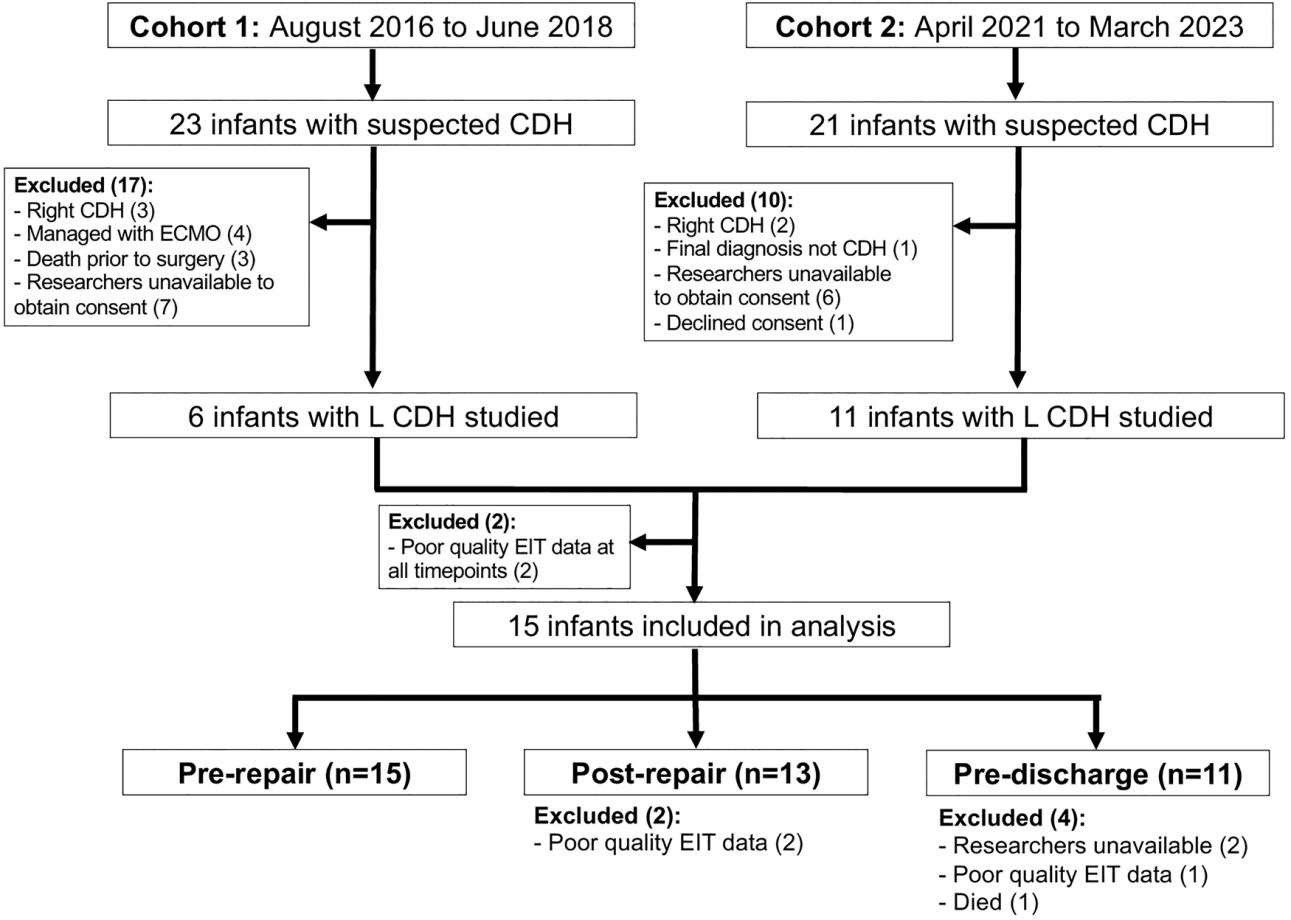
Participant inclusion flowchart. One infant whose data was excluded post-repair was receiving conventional mechanical ventilation and the other high-frequency oscillatory ventilation. The surviving three infants whose data were excluded pre-discharge were not receiving respiratory support for at least 5 days before discharge.

A total of 17 infants with left-sided CDH were studied, with two infants excluded for poor quality EIT data (Fig. 2**)**. Table 1 describes the clinical characteristics of included infants at each timepoint and further exclusions based on quality of imaging data. Due to requirements of stability during imaging, infants were more likely to have a lower-risk CDH profile.

**Table 1:** Clinical characteristics of included infants.

*n* = 15 infants			
**Gestational age at birth**	38 (34, 41) completed weeks		
**Birth weight**	3100 (2210, 3849) g		
**Antenatal diagnosis of CDH**	12 (80%)		
**Initial O/E LHR**	51 (36, 67) %		
**Liver in thorax antenatally**	5 (33%)		
**CDH Type**^40^	A 2^a^ (13%); B 9 (60%); C 4 (27%); D 0 (0%)		
**Patch required at repair**	4 (27%)		

Characteristics at each imaging timepoint			
	Pre-repair (*n* = 15)	Post-repair (*n* = 13)^b^	Pre-discharge (*n* = 11)^b^
**Age at recording, days**	3 (1, 6)	8 (5, 15)	24 (16, 123)
**Mode of ventilation**			
*High frequency oscillatory ventilation*	6 (40%)	5 (38%)	0
*High frequency jet ventilation*	1 (7%)	2 (15%)	0
*Conventional mechanical ventilation*	8 (53%)	5 (38%)	0
*Low flow oxygen*	0	0	1 (9%)
*No respiratory support*	0	1 (8%)	10 (91%)
**Ionotropic/lusitropic support**	14 (93%)	5 (38%)	0
**Pulmonary vasodilator**	9 (60%)	5 (38%)	3 (27%)
**Muscle relaxant infusion**	3 (20%)	1 (8%)	0

*CDH* Congenital diaphragmatic hernia, *O/E LHR* Observed to Expected Lung-to-Head Ratio.

Data presented as *n* (%) or median (minimum, maximum) unless otherwise stated.

^a^One infant found to have eventration of diaphragm at surgery.

^b^Includes only those infants with imaging data included in analysis (see Fig. 2).

### Distribution of tidal ventilation

**Fig. 3:**
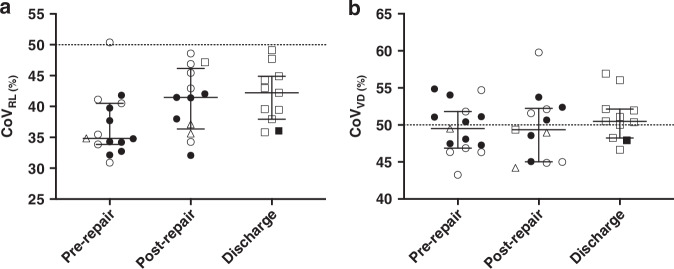
Distribution of ventilation. Centre of ventilation (CoV) in the right-left plane of the chest (**a**; CoV_RL_) and the gravity-dependent plane (**b**; CoV_VD_) for pre-repair, post-repair and pre-discharge time points. Symbols represent individual infant data (closed circle during conventional ventilation, open circles high frequency oscillatory ventilation [HFOV], open triangles high frequency jet ventilation [HFJV], closed square low flow oxygen and open square no respiratory support). Error bars median and interquartile range. 0 to 100% CoV represents right to left and gravity non-dependent to dependent, with 0% representing all ventilation in the most right and gravity non-dependent lung regions only (dashed line the value at true homogeneity of ventilation for EIT model used).

Overall, tidal ventilation was greater in the contralateral (right) chest at all timepoints (CoV_RL_ <50%) but increased in the ipsilateral (left) chest over the course of the NICU admission (Fig. 3A). Pre-discharge tidal ventilation had redistributed to the left lung by a median (95% CI) CoV_RL_ shift of 3.76 (0.39, 9.36)% from the pre-repair timepoint (Wilcoxon test). More infants with the largest documented antenatal O/E LHR ≥50% had CoV_RL_ > 40% (6/8 vs 1/5) post-repair, otherwise CoV_RL_ was similar (Supplementary Table S1 and Supplementary Fig. S2). Ventilation favoured the non-gravity dependent lung (ventral) at all timepoints but increased in the dependent lung by a median (95% CI) CoV_VD_ shift of 2.90 (−3.79, 5.81) from pre-repair to pre-discharge (Fig. 3B and Supplementary Table S2).

### Distribution of aeration

**Fig. 4:**
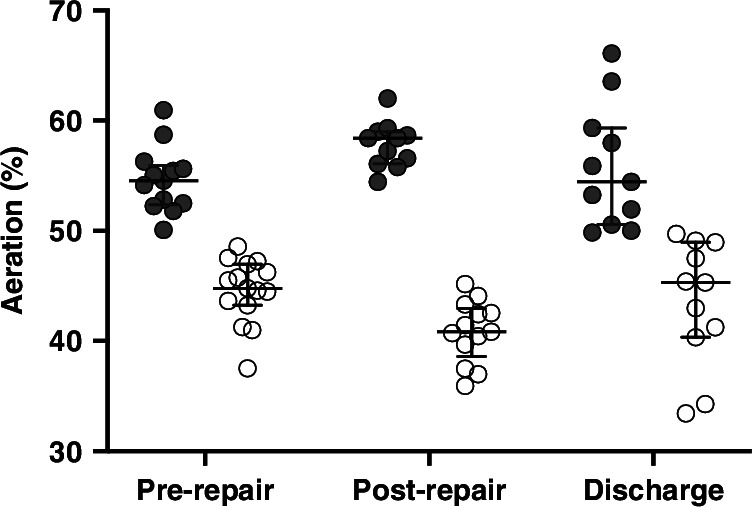
Contralateral (right; dark grey) and ipsilateral (left; light grey) lung aeration expressed as a percentage of whole lung aeration (end-expiratory lung volume) for pre-repair, post-repair and pre-discharge timepoints. Circles represent individual infant data. Error bars median and interquartile range. Supplementary Fig. S3 shows lung aeration data by mode of ventilation.

**Fig. 5:**
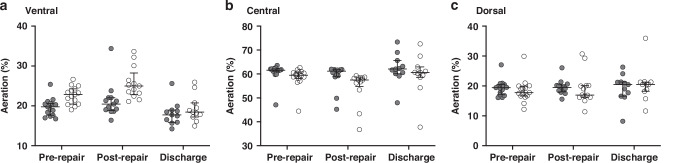
Gravity dependent distribution of contralateral (right; dark grey circles) and ipsilateral (left; light grey circles) regional lung aeration for pre-repair, post-repair and pre-discharge timepoints. Regional aeration presented for the ventral (**a**; most non-gravity dependent), central (**b**) and dorsal (**c**; most gravity dependent) thirds of the contralateral (right) and ipsilateral (left) lung expressed as a percentage of total lung aeration (end-expiratory lung volume) in each lung. Data presented as median and interquartile range, with circles representing individual infant values.

Relative aeration was greater in the contralateral (right) chest at all timepoints (Fig. 4 and Supplementary Table S3). Pre-repair and pre-discharge contralateral (right) and ipsilateral (left) aeration patterns were similar, but ipsilateral (left) aeration was lowest post-repair; median (range) 40.8 (35.9, 45.2)%. Gravity-dependent distribution of aeration was similar in the contralateral (right) and ipsilateral (left) chest at all timepoints (Fig. 5), with the most time-based changes between pre-repair and pre-discharge occurring in the ventral and dorsal regions of the ipsilateral lung.

### Mechanical characteristics of the contralateral (right) and ipsilateral (left) lung

Table 2 indicates the inspiratory and expiratory characteristics of the ipsilateral (left) and contralateral (right) lung during spontaneous breathing pre-discharge. Although there was no statistical difference, the contralateral (right) lung trended towards a faster inspiratory time constant and longer expiratory time.

**Table 2:** Mechanical characteristics of the contralateral (right) and ipsilateral (left) lung during spontaneous breathing before discharge home.

*n* = 11	Contralateral lung (Right)	Ipsilateral lung (Left)	Median (98.8% CI) difference
**T**_**i**_ **(ms)**	364 (271, 544)	365 (269, 550)	−3 (−6, 3)
**T**_**e**_ **(ms)**	401 (278, 685)	384 (264, 602)	7 (−26, 56)
**τ (ms)**	198 (143, 290)	203 (141, 292)	0 (−4, 3)

*T*_i_ inspiratory time, *T*_e_ expiratory time, *τ* inspiratory time constant (computed). Data presented as median (range), *n* = 11.

## Discussion

Due to the fine balance between the ventilation settings that deliver optimal support of gas exchange and those that are injurious, supporting the hypoplastic lungs of CDH is challenging.^3,9,10^ This may contribute to the relatively high rates of lung injury reported in infants with CDH.^8^ Arguably, the lack of reliable tools to define the magnitude and meaning of the differences in lung function both between lungs and over time has impeded CDH management. Our study is the first to describe lung function in both the ipsi- and contralateral lungs of infants born with CDH until NICU discharge. We found that regional ventilation and aeration were highly variable, but EIT was able to describe the expected patterns of right-left lung ventilation after surgery. The ability to repeatedly define regional ventilation characteristics offers the ability to better understand lung growth and function after NICU care and has the potential to guide early CDH management.

There was less ventilation in the ipsilateral (left) lung compared to the contralateral (right) lung. The pattern of right-left lung ventilation inhomogeneity in the pre-repair and post-repair timepoints was similar to that reported by Schroeder et al using a different EIT system in 12 infants with lower O/E LHR.^16^ The magnitude of inhomogeneity towards the contralateral lung was also greater than patterns reported in ventilated infants without CDH.^18^ This suggests that EIT can assess regional lung function in the anatomically different lungs of infants born with CDH. Radiological tools, such as chest radiograph and computerised tomography, are better for determining anatomical size.^12^ However, in states of heterogenous pulmonary hypoplasia, size may not reflect function. For example, an increase in anatomical volume may represent appropriate or abnormal lung growth or harmful overdistension.^29,30^ EIT is currently the best tool clinicians have to differentiate these states.^13^

The pattern of shift in CoV_RL_ towards the left lung immediately after surgery, and then more so at discharge, demonstrates that the ipsilateral CDH lung engaged in more ventilation with time. Immediately after surgery, this most likely represents the physical release of the compressed lung, an event that can create a high risk of injury from overdistension. The decrease in relative aeration suggests that gross overdistension was unlikely to be occurring in the infants we studied. As aeration was defined as relative to the whole lung, these changes indicated that aeration increased in the right lung without parallel changes in regional ventilation. This is reassuring regarding the ipsilateral lung but highlights that the contralateral lung is also vulnerable in CDH. Injury in each CDH lung has never been quantified in humans, and how best to ventilate lung disunity is unclear.^10,31^ The more complex nature of gas exchange during HFV (both HFOV and HFJV) may be advantageous in states of lung disunity.^32,33^ In our unit, HFOV and HFJV are used as rescue when conventional modes are not able to adequately support worsening cardiorespiratory failure. Even though V_T_ has been widely reported using EIT, it is possible that the much smaller V_T_ compared to CMV may alter EIT accuracy. Interestingly, we found that regional gas patterns did not cluster by mode of ventilation (although the subgroup numbers were small).

CDH is also characterised by abnormal pulmonary vasculature and right and left ventricular dysfunction.^2,3,34^ Improved ventilation may not translate to improved oxygenation unless matched with perfusion. Whilst EIT lacks the resolution to image pulmonary vascular development and ventricular function, cardiac-related EIT signals in the lungs during CMV have been reported as a representation of regional perfusion.^35^ This allows the potential to explore ventilation-perfusion matching in the CDH lungs in the future.

In people born with CDH, lung function requires life-long follow up due to the increased risk from routine infections of childhood, reflux-related aspiration and re-herniation,^36^ but beyond the immediate NICU period, it is poorly understood. To our knowledge, this is the first study to report lung function at NICU discharge in infants with CDH. Both ventilation and aeration tended to be greater pre-discharge, in the ipsilateral (left) lung compared to pre-repair. As these recordings were completed during spontaneous breathing rather than intubated support, and only four infants had a patch repair, lung growth is the most likely explanation. EIT also allowed examination of the waveform characteristics of volume (and thus flow) on a breath-by-breath basis.^12^ We identified only small differences in the T_i_ within the right and left lung during spontaneous breathing, and slightly greater differences in T_e_. These measurements were made prior to discharge when lung disunity may not have been as evident as immediately after birth. Due to the use of HFV and sedative agents, we were not able to accurately measure inspiratory and expiratory characteristics in the pre- and post-repair timepoints. As obstructive lung disease in late childhood is common in CDH survivors,^37,38^ we speculate that EIT measures of right-left lung T_e_ could be an early screening tool.

Although there were right-left lung differences in aeration, the gravity-dependent distribution was similar in each lung. This was unexpected and suggests a gravity-distribution pattern in each lung similar to that reported in normally developed preterm lungs.^18,19,28^ This is potentially important if replicated in larger studies. This suggests that despite the differences in pulmonary vasculature and alveolar development arising from pulmonary hypoplasia compared to the normally developed preterm lung, each of the CDH lungs may respond similarly to applied pressure, including recruitment manoeuvres, albeit cautiously, when atelectatic.

Our study has some limitations. Large studies in CDH have proved difficult and much of CDH management is guided by small physiological, observational studies with limitations.^10,27^ Ours is no exception. Reasonable conclusions regarding the relationship between EIT-derived lung function and known risk factors for the presumed magnitude of respiratory failure and hypoplasia in CDH such O/E LHR, right sided lesions, use of patch, ventilator pressure settings and gas exchange could not be made due to the small sample size.^10,34^ These relationships will need to be made to determine the utility of EIT in CDH management, especially in populations with lower O/E LHR, and would require multi-centre, international collaborations. Ideally, lung function should also be measured at more timepoints during the NICU stay. Our study was interrupted by the COVID-19 pandemic and skewed towards infants with less severe lung disease due to the need to handle the babies during high-risk clinical timepoints and follow-up to discharge. Despite this, our study and the recent study of Schroeder and co-workers demonstrate that EIT is feasible and tolerated in infants with CDH.^16^ The recent commercial availability of neonatal EIT units should allow increased use and familiarity. Importantly, the accuracy of EIT images depends on the mathematical models of the chest contents to define lung regions during image reconstruction.^12,22^ These models assume normal and consistent lung, heart and chest dimensions.^12,39^ The location and size of lungs and position of the heart in CDH is clearly not normal. As our study also demonstrated, it is likely highly variable. For this reason, we elected not to use a pre-existing infant chest model but simply define the chest by even chest regions and assume identified EIT changes were lung-related (by use of filtering). It is possible that lung tissue was not in the defined regions due to mediastinal shifts antenatally, or other gas containing tissue (i.e.,bowel) was present despite filtering.

## Conclusion

In this first study of lung function during NICU care in infants born with left-sided CDH, EIT was able to identify the expected right-left lung differences after birth. Importantly, we found evidence of ipsilateral improvements in function with time, suggestive of lung growth. Our study demonstrates the potential of EIT as a method of monitoring the CDH lung during NICU care and beyond.

### Data Statement

All data, including raw data used for all figures and analysis, and allocation of specific subjects in previous published material is available upon request to the corresponding author from three months following article publication to researchers who provide a methodologically sound proposal, with approval by an independent review committee (“learned intermediary”). Proposals should be directed to david.tingay@mcri.edu.au to gain access. Data requestors will need to sign a data access or material transfer agreement approved by MCRI.

## Supplementary information

Supplementary material

STROBE_checklist
