# Supplementary material for: Regional lung function in congenital diaphragmatic hernia assessed using electrical impedance tomography
Source: Pediatr Res. 2025 Jun 18;98(5):1773–9. doi: 10.1038/s41390-025-04185-9 (PMC12602320; doi:10.1038/s41390-025-04185-9)
Supplement: Supplementary file 1 — Supplementary material [file 41390_2025_4185_MOESM1_ESM.pdf]

Regional lung function in congenital diaphragmatic hernia assessed using electrical impedance tomography

Ellen Douglas[1*], Kristin N Ferguson[1,2*], David G Tingay[1,2,3,4]

*Joint first authors

1 Neonatal Research, Murdoch Children's Research Institute, Parkville, Australia

2 Neonatology, The Royal Children's Hospital, Parkville, Australia

3 Department of Paediatrics, University of Melbourne, Melbourne, Australia

4 Department of Critical Care, University of Melbourne, Melbourne, Australia

Supplementary Tables

Supplementary Table S1. Right to left lung Centre of ventilation (%) for each infant (rows) and time point presented by antenatal observed to expected lung head ratio (O/E LHR)

Supplementary Table S2. Ventral to dorsal lung Centre of ventilation (%) for each infant (rows) and time point presented by antenatal observed to expected lung head ratio (O/E LHR)

Supplementary Table S3. Left lung aeration (as a % of total lung aeration) for each infant (rows) and time point presented by antenatal observed to expected lung head ratio (O/E LHR)

Supplementary Figures

Supplementary Figure S1. Summary of study timeline.

Supplementary Figure S2. Centre of ventilation (CoV) in the right-left plane of the chest CoV_{RL} at the pre-repair, post-repair and pre-discharge time points for infants with an antenatal observed to expected lung head ratio <50% (black circles) or >50% (open diamonds).

Supplementary Figure S3. Contralateral (right; dark grey) and ipsilateral (left; light grey) lung aeration expressed as a percentage of whole lung aeration (end-expiratory lung volume) for pre-repair, post-repair and pre-discharge time points.

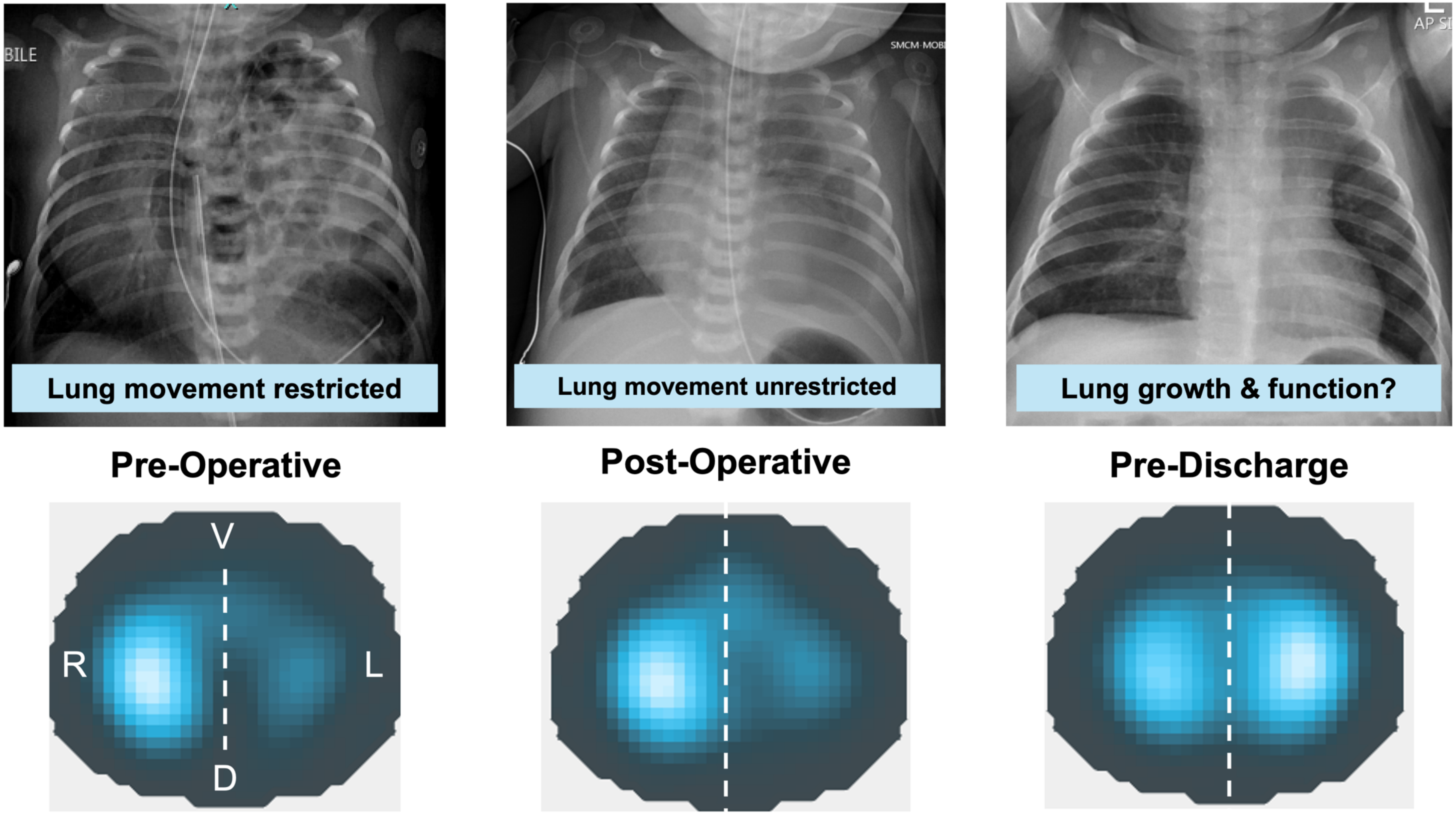
Lung movement restricted
Lung movement unrestricted
Lung growth & function?
Pre-Operative
Post-Operative
Pre-Discharge
V
R
L
D

Supplementary Figure S1. Summary of study timeline as per manuscript Figure 1 demonstrating representative functional electrical impedance tomography (EIT) images (no lung region filter) using the SD of the impedance within each pixel for all inflations analysed (represents the relative magnitude of V_T within each pixel).[1] Panels and labels otherwise as per manuscript Figure 1. Magnitude of V_T in each lung region is expressed using a colour scale from dark blue (minimum) to light blue/white (maximum). Abbreviations: R; right, L; left, V; ventral; D; dorsal (dotted line demarcates ipsilateral [left] and contralateral [right] hemithorax).[1]

Supplementary Table S1. Right to left lung Centre of ventilation (%) for each infant (rows) and time point presented by antenatal observed to expected lung head ratio (O/E LHR)

Pre-Repair	Post-Repair	Pre-Discharge
**O/E LHR > 50% (n=6 infants)**		
34.3	41.4	43.0
41.8	42.0	42.2
32.1	46.9	39.4
41.1	48.6	N/A
37.7	32.1	39.6
30.9	45.4	36.0
**O/E LHR < 50% (n=6 infants)**		
34.8	41.5	37.9
50.4	37.1	47.7
39.7	47.1	49.1
34.8	35.6	N/A
35.5	34.2	44.9
33.9	N/A	35.8
**No O/E LHR (Postnatal diagnosis; n=3)**		
32.7	N/A	N/A
34.2	38.0	N/A
40.5	42.9	44.3

Each row represents an infant

Colour of cell represents mode of ventilation: White - Conventional mechanical ventilation, Green - High-frequency oscillatory ventilation, Blue - High-frequency jet ventilation, Orange - Low flow nasal cannula, Yellow - Self-ventilating in air. N/A indicates no recording available.

Supplementary Table S2. Ventral to dorsal lung Centre of ventilation (%) for each infant (rows) and time point presented by antenatal observed to expected lung head ratio (O/E LHR)

Pre-Repair	Post-Repair	Pre-Discharge
**O/E LHR > 50% (n=6 infants)**		
54.0	48.6	52.0
48.1	45.1	50.5
54.9	45.0	52.1
51.8	52.1	N/A
51.1	52.4	56.9
54.7	44.9	47.9
**O/E LHR < 50% (n=6 infants)**		
47.5	53.7	50.4
46.3	44.2	50.0
50.4	49.4	46.6
49.5	49.0	N/A
43.3	59.8	48.2
46.9	-	56.0
**No O/E LHR (Postnatal diagnosis; n=3)**		
51.1	N/A	N/A
47.3	50.7	N/A
46.3	51.6	51.1

Each row represents an infant

Colour of cell represents mode of ventilation: White - Conventional mechanical ventilation, Green - High-frequency oscillatory ventilation, Blue - High-frequency jet ventilation, Orange - Low flow nasal cannula, Yellow - Self-ventilating in air. N/A indicates no recording available.

Supplementary Table S3. Left lung aeration (as a % of total lung aeration) for each infant (rows) and time point presented by antenatal observed to expected lung head ratio (O/E LHR)

Pre-Repair	Post-Repair	Pre-Discharge
**O/E LHR > 50% (n=6 infants)**		
45.8	40.4	45.3
43.2	45.2	43.0
46.2	37.5	49.1
47.5	41.4	N/A
41.3	35.9	41.3
44.8	39.7	34.3
**O/E LHR < 50% (n=6 infants)**		
45.5	42.4	40.4
44.5	42.6	49.7
47.3	44.1	33.4
41.0	40.7	N/A
44.6	37.0	49.0
47.0	N/A	45.4
**No O/E LHR (Postnatal diagnosis; n=3)**		
48.6	N/A	N/A
37.5	40.8	N/A
43.7	43.3	47.5

Colour of cell represents mode of ventilation: White - Conventional mechanical ventilation, Green - High-frequency oscillatory ventilation, Blue - High-frequency jet ventilation, Orange - Low flow nasal cannula, Yellow - Self-ventilating in air. N/A indicates no recording available.

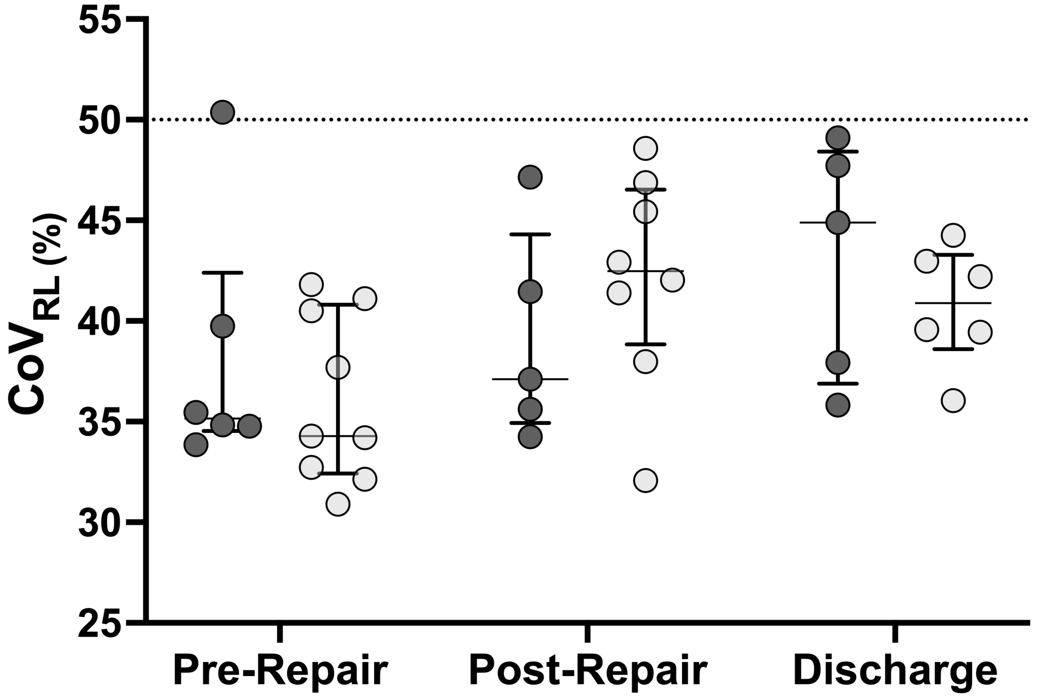

Supplementary Figure S2. Centre of ventilation (CoV) in the right-left plane of the chest CoV_{RL} at the pre-repair, post-repair and pre-discharge time points for infants with an antenatal observed to expected lung head ratio <50% (black circles) or >50% (open circles). Symbols represent individual infant data. Error bars median and interquartile range. Due to small sample size (and no pre-study subgroup analysis planned) no statistical analysis was performed. 0 to 100% CoV represents right to left and gravity non-dependent to dependent, with 0% representing all ventilation in the most right and gravity non-dependent lung regions only (dashed line the value at true homogeneity of ventilation). If more than one lung head ratio assessment was made during the pregnancy the greatest value was used.

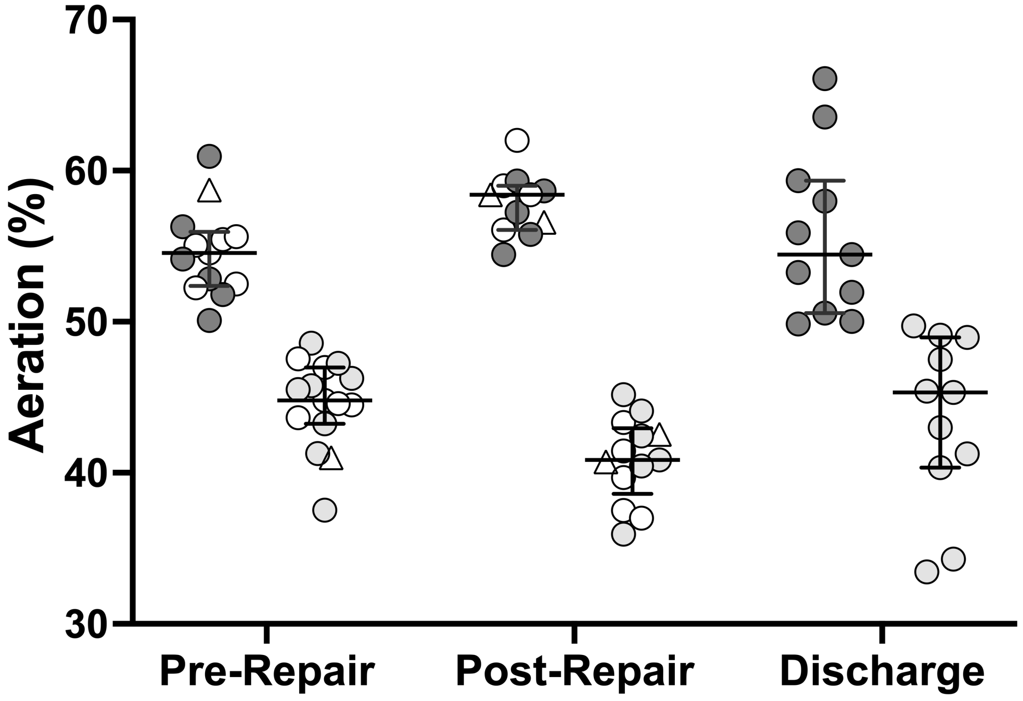

Supplementary Figure S3. Contralateral (right; dark grey) and ipsilateral (left; light grey) lung aeration expressed as a percentage of whole lung aeration (end-expiratory lung volume) for pre-repair, post-repair and pre-discharge time points. Symbols represent individual infant data: closed circle during conventional ventilation or low flow oxygen and open square no respiratory support (discharge); open circles high frequency oscillatory ventilation [HFOV], open triangles high frequency jet ventilation [HFJV]. Error bars median and interquartile range.

References

1. Frerichs, I.*, et al.* Chest electrical impedance tomography examination, data analysis, terminology, clinical use and recommendations: consensus statement of the TRanslational EIT developmeNt stuDy group. *Thorax* **72**, 83-93 (2017).
